# In Vitro Investigation of Six Antioxidants for Pig Diets

**DOI:** 10.3390/antiox5040041

**Published:** 2016-11-11

**Authors:** Hans Vergauwen, Sara Prims, Jeroen Degroote, Wei Wang, Christophe Casteleyn, Steven van Cruchten, Stefaan de Smet, Joris Michiels, Chris van Ginneken

**Affiliations:** 1 Laboratory of Applied Veterinary Morphology, Department of Veterinary Sciences, Faculty of Biomedical, University of Antwerp, Wilrijk 2610, Belgium; hans.vergauwen@uantwerpen.be (H.V.); sara.prims@uantwerpen.be (S.P.); christophe.casteleyn@uantwerpen.be (C.C.); steven.vancruchten@uantwerpen.be (S.v.C.); 2 Department of Applied Biosciences, Faculty of Bioscience Engineering, Ghent University, Ghent 9000, Belgium; jerdgroo.degroote@ugent.be (J.D.); Wei.Wang@nusciencegroup.com (W.W.); joris.michiels@ugent.be (J.M.); 3 Laboratory for Animal Nutrition and Animal Product Quality (LANUPRO), Department of Animal Production, Faculty of Bioscience Engineering, Ghent University, Melle 9090, Belgium; stefaan.desmet@ugent.be

**Keywords:** antioxidant pre-treatment, hydrogen peroxide, IPEC-J2 cells, small intestinal epithelial permeability, oxidative stress

## Abstract

Oxidative stress in the small intestinal epithelium can lead to barrier malfunction. In this study, the effect of rosmarinic acid (RA), quercetin (Que), gallic acid (GA), lipoic acid (LA), ethoxyquin (ETQ) and Se-methionine (SeMet) pre-treatments using 2 mM Trolox as a control on the viability and the generation of intracellular reactive oxygen species (iROS) of oxidatively (H_2_O_2_) stressed intestinal porcine epithelial cells (IPEC-J2) was investigated. A neutral red assay showed that RA (50–400 µM), Que (12.5–200 µM), GA (50–400 µM), ETQ (6.25–100 µM), and SeMet (125–1000 µM) pre-treatments but not LA significantly increased the viability of H_2_O_2_-stressed IPEC-J2 cells (*p* < 0.05). A 5-(and-6)-chloromethyl-2′,7′-dichlorodihydrofluorescein diacetate, acetyl ester (CM-H_2_DCFDA) fluorescent probe showed that RA (100–600 µM), Que (25–800 µM), ETQ (3.125–100 µM) and SeMet (500–2000 µM) pre-treatments significantly reduced iROS in IPEC-J2 monolayers (*p* < 0.05). Moreover, RA and Que were most effective in reducing iROS. Therefore, the effects of RA and Que on barrier functioning in vitro were examined. RA and Que pre-treatments significantly decreased fluorescein isothiocyanate (FITC)-conjugated dextran-4 (4 kDa) permeability and transepithelial electrical resistance (TEER) of an IPEC-J2 cell monolayer (*p* < 0.05). These in vitro results of RA and Que hold promise for their use as antioxidants in pig feed.

## 1. Introduction

Cellular lipids, proteins, and DNA structures can be damaged when the redox balance of the cell is disturbed by failure of the endogenous antioxidant system or the presence of excessive amounts of reactive oxygen species (ROS) [1]. Elevated levels of ROS in the mucosa of the jejunum are induced by changes in diet [2], and alter microbiota compositions [3,4]. Oxidation–reduction imbalance in the intestinal epithelium has an impact on its functionality [5]. The small intestine is in the primary site of nutrient absorption and part of a first line of defense in the body against dietary-derived oxidants [6,7]. Therefore, it is important to investigate how different types of antioxidants can be protective.

The inclusion of antioxidants in pig feed has various beneficial biological effects for piglet health [8]. Nonetheless, the antioxidant effects in the feed are well known; the detailed effects of dietary antioxidants at the level of the porcine intestinal tract remain unclear. Studying the functional effects of antioxidants on a porcine cell line mimicking the intestinal epithelium could substantiate and/or optimize the use of these antioxidants in pig feed. Thus far, in vitro research regarding antioxidants mainly used human carcinogenic intestinal epithelial cell lines [9,10]. In the present study, an intestinal porcine epithelial cell line isolated from the jejunum of a neonatal unsuckled piglet (IPEC-J2) was used. Previous research illustrated the link between oxidative stress and the functionality of porcine intestinal epithelial cells in vitro [11]. Porcine enterocyte survival and function were increased by 2 mM Trolox and 1 mM ascorbic acid pre-treatments [11]. The in vitro set-up allowed us to show that these beneficial effects were the result of the augmented intracellular antioxidant potential [11]. The aim of the present study was to document the effect of rosmarinic acid (RA), quercetin (Que), gallic acid (GA), lipoic acid (LA), ethoxyquin (ETQ) and Se-methionine (SeMet) on intracellular oxidative stress, and on the viability and functionality (integrity and barrier) of porcine small intestinal epithelial cells in vitro. The different compounds were selected after carefully revising available literature for their feasibility for effective implementation in swine nutrition. Moreover, they can be easily found in natural extracts, except for ETQ, which is a synthetic antioxidant.

## 2. Materials and Methods

### 2.1. Cell Line and Culture Conditions

IPEC-J2 cells (passages 60–65, ACC 701, DSMZ, Braunschweig, Germany) were cultured in DMEM/F-12 mix (Dulbecco’s modified Eagle medium, Ham’s F-12 mixture), 1.5 mM 4-(2-hydroxyethyl)-1-piperazineethanesulfonic acid (HEPES), 5% (v/v) fetal bovine serum, 1% (v/v) insulin-transferrin-selenium mixture, 1% (v/v) penicillin-streptomycin mixture and 2.5 µg/mL fungizone (Invitrogen, Merelbeke, Belgium) (37 °C, 5% CO_2_). The culture medium was refreshed every other day. When studying the permeability, IPEC-J2 cells were seeded (1 × 105 cells/well) at confluence in a “Boyden chamber” insert (upper chamber, apical) on a polyethylene terephthalate (PET) membrane (1.12 cm^2^, pore size 0.4 µm, ThinCert^®^, Greiner Bio-One, Vilvoorde, Belgium). To measure the amount of permeated FD-4, culture medium without phenol red was used to culture the IPEC-J2 cells. When investigating intracellular oxidative stress, cells were seeded in a 12-well plate (1 × 105 cells/well, flat bottom, Greiner Bio-One). Cells were seeded in a 96-well plate (0.5 × 104/well, flat bottom, Greiner Bio-One) to assess viability.

### 2.2. Induction of Oxidative Stress

To induce oxidative stress, cells were incubated with H_2_O_2_ (0.5 or 1 mM, Sigma-Aldrich, Overijse, Belgium), which was added to the apical chamber for 1 h. The stressor was removed by washing twice with DMEM/F-12 mix without additives (washing buffer). The different concentrations of H_2_O_2_ for each specific experiment have been previously optimized, 0.5 mM for the intracellular oxidative stress assay, and 1 mM for the viability and permeability assay [11].

### 2.3. Antioxidant Pre-Treatments

Cells were pre-treated (overnight, 18 h) with 12.5–1600 µM rosmarinic acid (RA), 25–800 µM quercetin (Que), 12.5–800 µM gallic acid (GA), 12.5–800 µM lipoic acid (LA), 3.125–100 µM ethoxyquin (ETQ) and 4–2000 µM Se-methionine (SeMet) (Sigma-Aldrich). During all experiments, the soluble form of vitamin E, Trolox (2 mM), was used as a control antioxidant [11]. All antioxidants were dissolved in culture medium and filtered (0.2 µm, Acrodisc^®^ Syringe Filters with HT Tuffryn^®^ Membrane, PALL, New York, NY, USA) before application on the IPEC-J2 cells. Que and ETQ were dissolved in culture medium with 0.05% dimethylsulfoxide (DMSO).

### 2.4. Viability (Neutral Red)

After treatment with or without antioxidants, the cells were washed twice with washing buffer and subsequently incubated in the presence (test group) or absence (control group) of 1 mM H_2_O_2_ for 1 h and washed twice again. After removing the culture medium, 100 µL washing buffer plus 50 µL 0.01% (w/v) neutral red dye (Janssen Chimica, Beerse, Belgium) was added to each well, and incubated for 2 h (37 °C, 5% CO_2_). Afterwards, the cells were washed twice with phosphate-buffered saline (PBS, pH 7.4, 0.01 M) to remove the neutral red dye that was not incorporated by the lysosomes. Subsequently, the dye was extracted from the cells using 100 µL of 50% (v/v) ethanol solution (in 0.05 M NaH_2_PO_4_). The absorbance was measured at 550 nm using a spectrophotometer (Infinite^®^ M200 PRO, Tecan, Männedorf, Switzerland). The viability data are presented as a percentage of the control, and thus unstressed cells are 100% viable.

### 2.5. Intracellular Oxidative Stress

The intracellular oxidative stress was analyzed using a ROS-sensitive probe, i.e., 5-(and-6-)-chloromethyl-2′,7′-dichlorodihydrofluorescein diacetate, acetyl ester (CM-H_2_DCFDA) (Ex/Em: 492–495/517–527 nm) (Invitrogen, Merelbeke, Belgium). The probe detects intracellular H_2_O_2_ and downstream products. After treatment with or without antioxidants, the cells were washed twice with washing buffer, incubated for 30 min with 750 µL of 8.67 µM CM-H2DCFDA (diluted in washing buffer), and washed twice. Subsequently, the cells were incubated in the presence (test group) or absence (control group) of 0.5 mM H_2_O_2_ for 1 h, washed twice and finally the culture medium was refreshed and left to equilibrate for 1 h. The cells were trypsinized, and washed twice with PBS including a centrifugation step (400× *g*, 5 min). The cells were analyzed using a flow cytometer (Coulter EPICS XL-MCL, Suarlée, Belgium). The viability was assessed by the addition of 1 µL GelRed (VWR, Leuven, Belgium) per mL cell suspension. The control consisted of unstressed cells incubated solely with ROS detection probe to account for the ROS generated by the normal/basal energy metabolism of the cell. All analyses were performed according to flow cytometry standard procedures, and, for each sample, at least 10,000 cells were measured. Flow cytometric data analysis was performed using FlowJo version 10.0.6 (Treestar, Ashland, OR, USA). The data obtained after flow cytometric analysis was presented as mean fluorescence intensity (MFI), which are arbitrary units.

### 2.6. Permeability Assay

After treatment with or without antioxidants, the cells were washed twice and incubated in the presence (test group) or absence (control group) of 1 mM H_2_O_2_ for 1 h. The cells were washed twice again. The permeability assay started when 500 µL culture medium without phenol red containing 50 µg FITC-conjugated dextran-4 (FD-4, 4 kDa) (Sigma-Aldrich) was added to the apical chamber. The basolateral chamber was filled with 1.5 mL culture medium without phenol red (37 °C, 5% CO_2_). The FD-4 was allowed to permeate overnight (18 h) from the apical to basolateral chamber. Subsequently, 100 µL of the basolateral chamber was transferred to a 96-well plate. The amount of permeated FD-4 was measured using a fluorospectrophotometer (Ex/Em: 490/520 nm, Infinite^®^ M200 PRO, Tecan, Männedorf, Switzerland). The acquired data were processed using i-control^TM^ data analysis software (version 1.9, Infinite^®^ M200 PRO, Tecan, Männedorf, Switzerland). The permeability data was presented as picomoles of FD-4 that permeated through the IPEC-J2 cell monolayer into the basolateral chamber.

### 2.7. Transepithelial Electrical Resistance (TEER)

When the IPEC-J2 monolayer was confluent (≥1 kΩ·cm^2^), the cells were pre-treated with or without antioxidants for 18 h. The cells were washed twice and incubated with an oxidative stressor for 1 h. The oxidative stressor was removed by washing the cells twice. The cells were incubated overnight with culture medium to achieve stable TEER values. TEER was determined using a Millicell-ERS (electrical resistance system) with STX01 electrodes (Millipore, Overijse, Belgium) and calculated as kΩ·cm^2^ by multiplying by the surface area of the monolayer (1.2 cm^2^). Since TEER was quickly affected by changes in temperature, pH and washing steps, data were only collected after a stabilisation period of 1 or 2 days. The baseline resistance of a ThinCert porous membrane was approximately 50 Ω·cm^2^. This value was subtracted from all TEER data.

### 2.8. Statistical Analysis

Data are presented as estimated means ± standard error (S.E.) (*n* = 2, with 6 replicates within an experiment), with n referring to the number of experiments repeated in time JMP Pro 11 (JMP software, SAS, Tervuren, Belgium) was used for statistical evaluation. Normality of the distribution was evaluated by the Shapiro–Wilk test and the homogeneity of variances was analyzed by the Levene’s test. Due to the heterogeneity of the variances, the data were analyzed using the non-parametric Kruskal–Wallis test followed by post hoc testing (Mann–Whitney U test). A *p* value of <0.05 was considered statistically significant.

## 3. Results

### 3.1. Effect of Antioxidants on the Viability of 1 mM H_2_O_2_ Stressed IPEC-J2 cells

**Figure 1:**
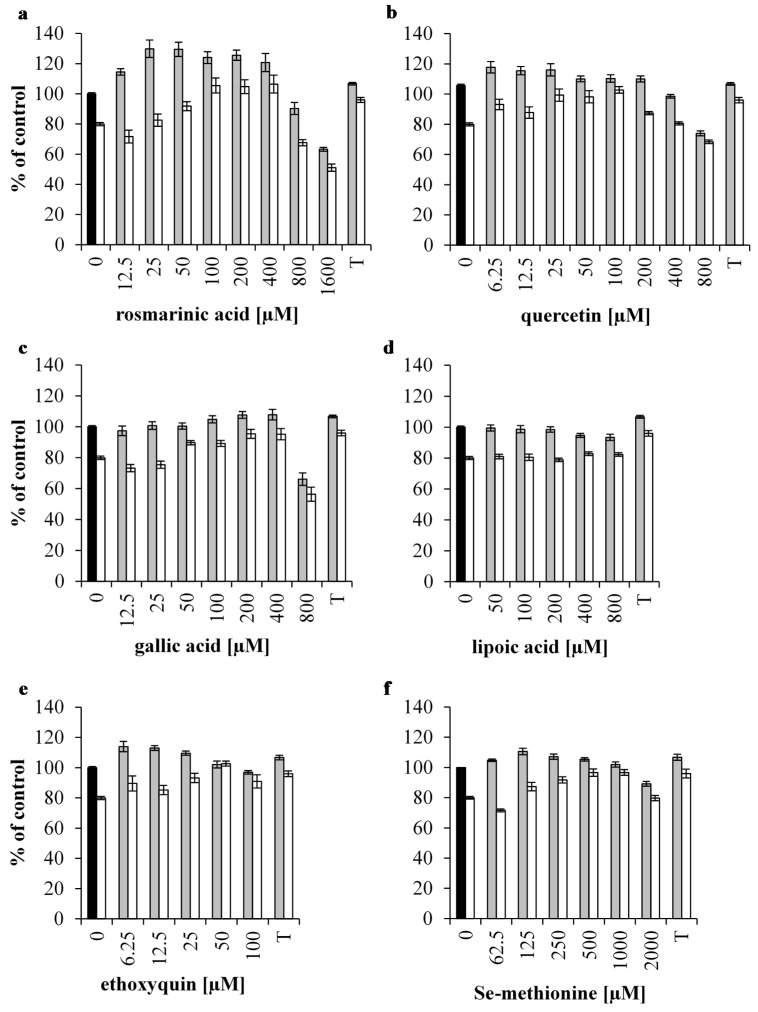
Viability of H_2_O_2_ treated intestinal porcine epithelial cells (IPEC-J2) pre-treated with antioxidants. Viability of IPEC-J2 cells is presented as a percentage of the untreated control cells (**black** bar: untreated control cells, **grey** bars: no incubation with oxidative stress; **white** bars: cells stressed by 1 mM H_2_O_2_) after treatment with (**a**) rosmarinic acid (RA), (**b**) quercetin (Que), (**c**) gallic acid (GA), (**d**) lipoic acid (LA), (**e**) ethoxyquin (ETQ) and (**f**) selenium-methionine (SeMet). All antioxidant pre-treatments were compared with 2 mM Trolox (T). Results are presented as means ± S.E., *n* = 2.

The viability is represented as a percentage of control, with the viability of the untreated cell being 100%. The viability of the cells incubated with 1 mM H_2_O_2_ for 1 h was significantly reduced to 80% (*p* < 0.001). Incubating IPEC-J2 cells with 12.5, 25, 50, 100, 200 and 400 µM RA significantly increased viability compared to untreated cells (*p* < 0.05) and 2 mM Trolox (*p* < 0.01) (Figure 1a). For several concentrations of RA, the viability rose over 100%. Depending on the experimental set-up, it is not uncommon that the viability increases over 100%, as test samples are compared to a control sample and not to an internal control, as such washing steps and the specific cell density at the start of the experiment could explain these differences [12]. Incubating with high concentrations such as 800 and 1600 µM RA significantly decreased viability to, respectively, 90% and 63% of the unstressed cells (*p* < 0.05). The viability of 1 mM H_2_O_2_ treated cells was significantly increased by 50, 100, 200 and 400 µM RA pre-treatments (*p* < 0.001). All of these RA pre-treatments, except 50 µM RA, were able to restore viability to untreated control levels. Only the 200 µM RA pre-treatment was able to significantly increase the viability of oxidatively stressed cells compared to the 2 mM Trolox pre-treatment (*p* < 0.05). No significant difference was found between 100, 200 and 400 µM RA pre-treatments in oxidatively stressed cells (*p* > 0.05). Furthermore, pre-incubation with 400 µM RA in H_2_O_2_ treated cells showed no difference in viability compared to its non-oxidatively stressed control levels (*p* > 0.05).

When the cells were incubated with 6.25, 12.5, 25, 50, 100 and 200 µM Que the viability of unstressed IPEC-J2 cells rose (*p* < 0.001) (Figure 1B). Concentrations ranging between 6.25–100 µM Que significantly increased the viability compared to 2 mM Trolox (*p* < 0.05). Treatment of unstressed IPEC-J2 cells with 400 µM Que had no significant effect on the viability of cells, while 800 µM Que significantly decreased the viability (*p* < 0.001). Both 400 and 800 µM were significantly less effective than 2 mM Trolox (*p* < 0.0001). The viability of 1 mM H_2_O_2_ treated cells was significantly increased by 12.5, 25, 50, 100 and 200 µM Que pre-treatments (*p* < 0.05). Of these concentrations, only 25, 50 and 100 µM Que pre-treatments were able to restore viability of the H_2_O_2_ treated cells to untreated control and to the 2 mM Trolox pre-treatment levels (*p* > 0.05). Cells that were pre-incubated with 50 or 100 µM Que and subsequently treated with H_2_O_2_ restored their viability to their respective non-oxidatively stressed control levels (*p* > 0.05).

Cells that were incubated with 100, 200 and 400 µM GA had a significantly higher viability (*p* < 0.05), but to a similar extent as when incubated with 2 mM Trolox (*p* > 0.05) (Figure 1b). Concentrations below 100 µM GA (e.g., 12.5, 25 and 50 µM) did not affect the viability of IPEC-J2 cells. Furthermore, 800 µM GA significantly decreased the viability (*p* < 0.001). The viability of H_2_O_2_ treated cells was significantly restored to untreated control levels by 50, 100, 200 and 400 µM GA pre-treatments. None of the GA pre-treatments was able to increase the viability to their respective non-oxidatively stressed control levels (*p* < 0.05). Furthermore, no significant difference was found when 200 and 400 µM GA pre-treatments were compared to the 2 mM Trolox pre-treatment (*p* > 0.05) (Figure 1c).

Incubation with 400 and 800 µM LA significantly decreased the IPEC-J2 viability (*p* < 0.05), while all other LA concentrations (e.g., 50, 100 and 200 µM) had no significant influence on the viability of IPEC-J2 cells (Figure 1d).

Incubation with 6.25, 12.5 and 25 µM ETQ significantly increased IPEC-J2 viability (*p* < 0.001) (Figure 1e), while 50 and 100 µM ETQ treatments had no significant effect (Figure 1d). Unstressed cells incubated with 3.25 and 12.5 µM ETQ had a significantly higher viability than 2 mM Trolox treated cells (*p* < 0.05), whereas 50 and 100 µm ETQ showed a significantly lower viability when compared with 2 mM Trolox (*p* < 0.01). Nevertheless, all ETQ concentrations significantly increased the viability of oxidatively stressed cells (*p* < 0.05). Cells pre-treated 25, 50 and 100 µM ETQ and subsequently treated with H_2_O_2_ showed no significant difference of viability compared to their respective non-oxidatively stressed controls (*p* > 0.05). However, only 50 µM ETQ pre-treatment in oxidatively stressed cells was able to restore the viability to untreated control levels and proved to be more effective than the 2 mM Trolox pre-treatment (*p* < 0.01) (Figure 1e).

Cells incubated with concentrations of SeMet ranging between 62.5–500 µM showed an increased viability (*p* < 0.05), and were not different from 2 mM Trolox (*p* > 0.05) (Figure 1f). Incubation with 1000 µM SeMet had no significant effect on the viability, whereas 2000 µM SeMet treatment significantly decreased the viability (*p* < 0.001). In cells that were oxidatively stressed, 125, 250, 500 and 1000 µM SeMet pre-treatments restored the viability (*p* < 0.05). No significant difference in viability was found between 250, 500 and 1000 µM SeMet pre-treatments of oxidatively stressed cells when compared to the 2 mM Trolox pre-treatment (*p* > 0.05). In contrast to RA, Que, and ETQ, none of the SeMet pre-treatments were able to increase the viability to their respective non-oxidatively stressed control levels.

### 3.2. Effect of Antioxidants on Intracellular Oxidative Stress of 0.5 mM H_2_O_2_ Treated IPEC-J2 Cells

**Figure 2:**
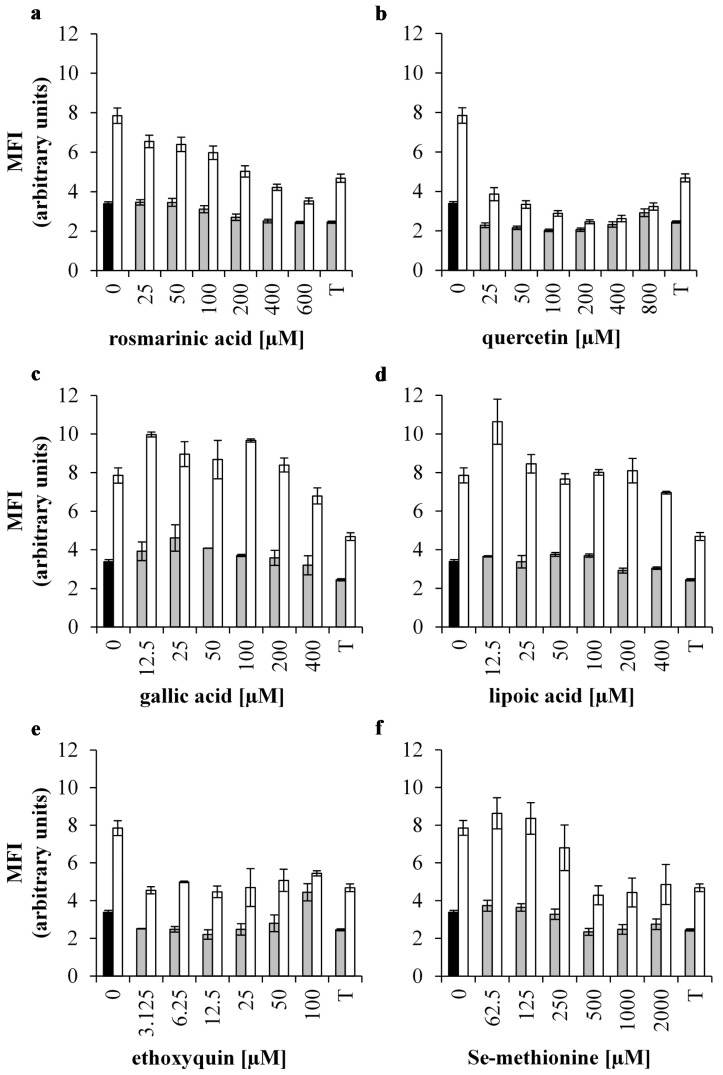
Intracellular oxidative stress of H_2_O_2_ treated IPEC-J2 cells pre-treated with antioxidants. Generation of intracellular reactive oxygen species (iROS) is reported as mean fluorescent intensity (MFI) and measured in unstressed and H_2_O_2_ treated IPEC-J2 cells (**black** bar: untreated control cells, **grey** bars: no incubation with oxidative stress; **white** bars: cells stressed by 1 mM H_2_O_2_) after treatment with (**a**) RA, (**b**) Que, (**c**) GA, (**d**) LA, (**e**) ETQ and (**f**) SeMet. All antioxidant pre-treatments were compared with 2 mM Trolox (T). Results are presented as means ± S.E., *n* = 2.

Incubating IPEC-J2 cells with 0.5 mM H_2_O_2_ significantly increased the amount of intracellular reactive oxygen species (iROS) (*p* < 0.001). Pre-incubating the cells with 2 mM Trolox significantly decreased the generation of iROS under control conditions and when the IPEC-J2 cells were challenged with 0.5 mM H_2_O_2_ (*p* < 0.001) (Figure 2a–f).

Incubating cells with 200, 400 and 600 µM RA significantly decreased iROS in control conditions as well as in oxidatively stressed cells (*p* < 0.05). Concentrations below 200 µM RA had no effect (Figure 2a). The effect of 200, 400 and 600 µM RA on iROS production was similar to the effect of 2 mM Trolox (*p* > 0.05). The highest concentration of RA (600 µM) was more potent than 2 mM Trolox pre-treatment to significantly reduce iROS (*p* < 0.01).

Que significantly decreased iROS production in unstressed IPEC-J2 cells (*p* < 0.001) (Figure 2b). Moreover, 50, 100 and 200 µM Que resulted in iROS levels that were significantly below the levels of 2 mM Trolox (*p* < 0.05). When IPEC-J2 cells were stressed with 0.5 mM H_2_O_2_, all Que pre-treatments significantly decreased iROS (*p* < 0.001), and proved to be more effective than 2 mM Trolox (*p* < 0.05). Cells that were pre-incubated with 400 or 800 µM Que and subsequently treated with H_2_O_2_ decreased iROS to their respective non-oxidatively stressed control levels (*p* > 0.05). However, 200 µM Que pre-treatment showed to be the most effective in oxidatively stressed IPEC-J2 cells (*p* < 0.001).

GA concentrations above 50 µM had no effect on iROS production (*p* > 0.05), whereas 12.5, 25 and 50 µM GA significantly increased the amount of iROS in unstressed IPEC-J2 cells (*p* < 0.05) (Figure 2c). Pre-treatment with 25, 50, 200 and 400 µM GA did not affect iROS in 0.5 mM H_2_O_2_ treated cells (*p* > 0.05) and because of the negligible antioxidant effects, GA was excluded from the permeability assay. 

LA incubation of 12.5–400 µM was not affected (*p* > 0.05) or even increased (*p* < 0.05) iROS production (Figure 2d) in both normal and oxidatively stressed cells. Therefore, LA was excluded from the permeability assay.

iROS was significantly decreased in cells that were incubated with 3.125, 6.25, 12.5, 25 or 50 µM ETQ (*p* < 0.05) (Figure 2e). In contrast, incubation with 100 µM ETQ increased iROS levels (*p* < 0.01). All ETQ pre-treatments significantly decreased the excessive iROS production caused by H_2_O_2_ (*p* < 0.05). The anti-oxidative effect of ETQ comparable to the reduction seen when pre-incubated with 2 mM Trolox (*p* > 0.05). Therefore, ETQ was excluded from the permeability assay.

Only 500 and 1000 µM SeMet treatments decreased iROS in unstressed IPEC-J2 cells (*p* < 0.05) (Figure 2f). These results are comparable to what was seen when treated with 2mM Trolox (*p* > 0.05). When cells were oxidatively stressed, only 500, 1000 and 2000 µM SeMet pre-treatments significantly decreased iROS (*p* < 0.05) to an extent that is comparable with 2 mM Trolox (*p* > 0.05). Therefore, SeMet was excluded from the permeability assay.

### 3.3. Protective Effect of Quercetin and Rosmarinic Acid on the Permeability and Integrity of A 1 mM H_2_O_2_ Treated IPEC-J2 Monolayer

**Figure 3:**
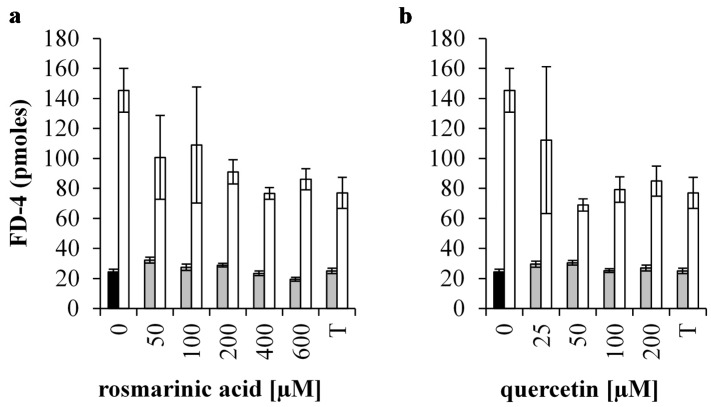
Membrane permeability of H_2_O_2_ treated IPEC-J2 cells pre-treated with antioxidants. The permeability of the monolayer is reported as the amount of FD-4 (pmoles) measured in the basal compartment of the Boyden chamber of unstressed and H_2_O_2_ treated IPEC-J2 cells (**black** bar: untreated control cells, **grey** bars: no incubation with oxidative stress; **white** bars: cells stressed by 1 mM H_2_O_2_) after treatment with (**a**) RA and (**b**) Que. Both antioxidant pre-treatments were compared with 2 mM Trolox (T). Results are presented as means ± S.E., *n* = 2.

The effect on the FD-4 permeability and TEER was investigated for RA and Que pre-treatments. A disruption of the IPEC-J2 monolayer by 1 mM H_2_O_2_ was expected as the amount of permeated FD-4 increased approximately six-fold (*p* < 0.001) (Figure 3a,b). Pre-incubation with 2 mM Trolox was able to partially preserve the integrity of the monolayer when cells were stressed with H_2_O_2_ (*p* < 0.01).

Treatments with 100, 200, 400 and 600 µM RA did not affect FD-4 fluxes (*p* > 0.05) (Figure 3a). Treatment with 50 µM RA slightly increased FD-4 flux as compared to the control values (*p* < 0.05). Under conditions of oxidative stress (treatment with 1 mM H_2_O_2_) pre-incubation with 200, 400 and 600 µM RA FD-4 fluxes were significantly lower (*p* < 0.05). These effects were comparable with the effect when the cells were pre-treated with 2 mM Trolox (*p* > 0.05).

Incubation with 25, 50, 100 and 200 µM Que had no effect on permeability under unstressed conditions (*p* > 0.05) (Figure 3b). The integrity of the monolayer that was challenged by 1 mM H_2_O_2_ was significantly preserved by all Que pre-treatments (*p* < 0.05) to the same extent as when pre-incubated with 2 mM Trolox (*p* > 0.05).

**Figure 4:**
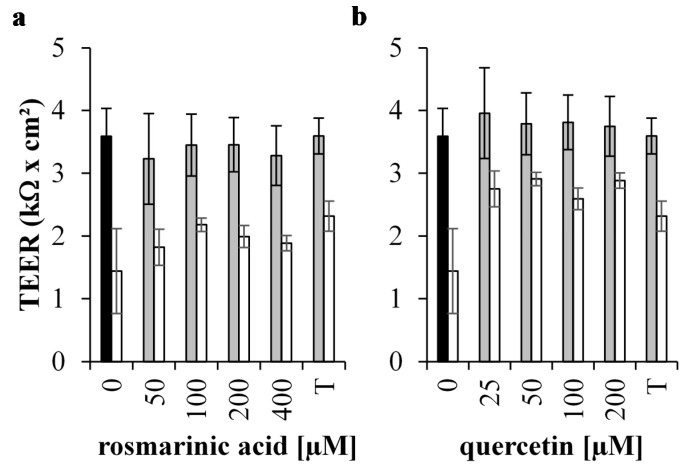
Membrane integrity of H_2_O_2_ treated IPEC-J2 cells pre-treated with antioxidants. The integrity of the monolayer is reported as the amount TEER (kΩ·cm^2^) measured between the basal and apical compartment of the Boyden chamber of unstressed and H_2_O_2_ treated IPEC-J2 cells (**black** bar: untreated control cells, **grey** bars: no incubation with oxidative stress; **white** bars: cells stressed by 1 mM H_2_O_2_) after treatment with (**a**) RA and (**b**) Que. Both antioxidant pre-treatments were compared with 2 mM Trolox (T). Results are presented as means ± S.E., *n* = 2.

The integrity of the IPEC-J2 cell monolayer was compromised by 1 mM H_2_O_2_ as TEER dropped significantly compared to the control (*p* < 0.001) (Figure 4a,b).

Incubation with RA (50–400 µM) or with Que (25–200 µM) had no effect on the TEER of the IPEC-J2 monolayer (*p* > 0.05). Pre-incubation with 2 mM Trolox (*p* < 0.01), 100 µM RA (*p* < 0.05) or 25–200 µM Que (*p* < 0.01) partially preserved the TEER of 1 mM H_2_O_2_ treated IPEC-J2 monolayers. TEER with these pretreatments was still lower than under control conditions (*p* < 0.01). Only Que pre-treatments were able to preserve the TEER more efficiently compared to 2 mM Trolox pre-treatment (*p* < 0.05).

## 4. Discussion

ROS fulfill specific functions in a wide variety of signal-transduction pathways in both gastrointestinal health and disease [13]. ROS production increases in a pig upon weaning resulting in oxidative stress [14]. The latter can contribute to the increased disease susceptibility and mortality that is seen in relation to weaning [15]. Intracellular reactive oxygen species (iROS) are highly reactive molecules that can cause tissue damage by reacting with polyunsaturated fatty acids in cellular membranes, nucleotides in DNA, and critical sulfhydryl bonds in proteins [16]. The intricate balance between sufficient ROS for normal functioning versus excessive ROS production leading to oxidative stress is controlled by the endogenous antioxidant defense system comprising glutathione, glutathione peroxidase, peroxiredoxins, superoxide dismutase and catalase [17,18,19]. Besides this endogenously regulated system, support is possible via exogenous antioxidants such as vitamin E, vitamin C, polyphenols, and carotenoids [13,20]. Therefore, our study focused on the investigation of promising antioxidants and their ability to reduce intracellular oxidative stress in porcine small intestinal enterocytes.

Ma et al., demonstrated the importance of IPEC-J2 cells to study dietary antioxidants prior to supplementation to piglet feed in the pig industry [21]. We previously optimized the IPEC-J2 oxidative stress model to link effects of oxidative stress with functional outcome measures such as regeneration potential and barrier functioning [11]. The present study used the IPEC-J2 oxidative stress (H_2_O_2_) model to test different antioxidants. Pre-treatments of RA, Que, GA, ETQ and SeMet improved the survival of porcine enterocytes that were challenged with H_2_O_2_. The beneficial effect on porcine cell survival of these antioxidants can be related to their specific structures and confirms previous human and bovine in vitro research e.g., for RA [22], Que [23], GA [24], ETQ [25] and SeMet [26]. These antioxidants, except SeMet, possess one or more aromatic rings and/or double bonds that increase the efficacy of receiving electrons through resonance in order to stabilize free radicals after donating a proton to the ROS [27]. Additionally, the selenium in SeMet can stabilize electrons through its strong nucleophilic properties. The electron cloud of selenium is larger and the valence electrons are further away from the positive core compared to sulphur, and therefore it is more readily oxidized than sulphur [28].

The results in this study demonstrated that moderate to low concentrations of RA, Que, GA, ETQ, and SeMet improved cell viability in non-carcinogenic, non-transformed unstressed porcine intestinal cells. However, high concentrations of RA reduced viability. A similar decrease of viability was seen in Caco-2 cells when incubated with rosemary extract and could be attributed to a cytotoxic effect of RA [9]. Similarly, treatment with 100 µM Que has been shown to induce DNA damage in Caco-2 cells [29]. This concentration of Que was not deemed cytotoxic in our IPEC-J2 cells. Furthermore, anti-proliferative activities of GA were observed in Caco-2, HepG2, MCF-7, and WT-F344 rat liver cells using the 3-(4,5-dimethylthiazolyl-2)-2,5-diphenyltetrazolium bromide (MTT) assay and were suggested to be caused by the pro-oxidative effects of GA [30]. Thus, GA shows selective cytotoxicity for cancerous human cells, but, in our setting, very little toxicity for normal porcine cells. Thus, the use of non-carcinogenic, non-transformed intestinal epithelial cell lines is favored.

Cai et al., reported that 10 µg/mL LA significantly increased the cytoactivity (MTT assay) of H_2_O_2_-stressed IPEC-J2 cells [31]. This differs from our findings where we observed no effect on cell viability of LA. Blaszczyk and Skolimowski reported a significant decrease of human peripheral lymphocyte viability after 24 h treatment with 250 µM ETQ [32]. Similar to our study, no effect was observed when the lymphocytes were treated with 100 µM ETQ. SeMet treatments have been shown to exert p53 dependent growth inhibitory effects in colon cancer cells by inducing G2/M cell cycle arrest as well as apoptosis [33]. On the other hand, SeMet enhances expression of selenoproteins and the viability of non-carcinogenic bovine mammary epithelial cells, treated with or without H_2_O_2_ [26]. Taken together, these reports show that antioxidants aid cell survival in the presence of oxidative stress in both normal and carcinogenic cell lines. However, in the absence of a ROS inducer, antioxidants commonly decrease cell proliferation.

This study showed that RA, Que, ETQ, and SeMet significantly reduced intracellular oxidative species in H_2_O_2_ stressed IPEC-J2 cells. Only RA and Que were able to completely restore the iROS concentrations to the unstressed control levels. Previously, RA has been shown to inhibit linoleic acid peroxidation and its antioxidant activity noticeably exceeds that of Trolox in Hb-H_2_O_2_-luminol and 2,2′-azobis (2-methylpropionamidine) dihydrochloride-luminol systems [34]. It was shown that RA attenuated cellular oxidative stress by decreasing the amount of iROS and malondialdehyde in H_2_O_2_ treated astrocytes [22]. Yokomizo and Moriwaki observed that Que was able to reduce iROS production in H_2_O_2_ treated Caco-2 cells and reduce iROS below control values in Caco-2 cells that were oxidatively stressed by linoleic acid hydroperoxide [35]. In our experimental set-up, ETQ and SeMet pre-treatments only partially decreased intracellular oxidative stress. Miranda et al. observed decreased iROS levels in H_2_O_2_ treated bovine mammary epithelial cells when pre-incubated with SeMet [26]. Finally, GA and LA pre-treatments were unable to significantly decrease the intracellular oxidative stress in IPEC-J2 cells. Previous research showed that LA pre-treatment had the tendency to decrease iROS in IPEC-J2 cells; however, this was not significant [31]. This study showed that GA increased the viability of H_2_O_2_ stressed cells but was unable to significantly decrease iROS. GA has been shown to have a variety of cellular effects. It can deplete intracellular glutathione and thereby increase iROS or simply act as a pro-oxidant [36]. On the other hand, GA pre-treatments significantly decreased iROS generation in HeLa cells [37]. These differing effects are not uncommon, as polyphenolics can exhibit antioxidants as well as pro-oxidant activities depending on factors such as bioavailability and stability in tissue [38].

The small intestine serves primarily as an absorptive organ, and also metabolizes nutrients, drugs and other molecules. Besides the absorptive and metabolic capabilities, the intestinal epithelium forms a physical barrier that acts as a functional line of defense against toxins and other insults [6]. One of the pathophysiological mechanisms responsible for barrier dysfunction is oxidative stress. H_2_O_2_ and superoxide radical have been shown to increase the permeability in epithelial monolayers by disrupting the cytoskeleton [39]. It has been established that tight junctions reorganize due to the effects of oxidative stress, and, as a consequence, that TEER values of cell monolayers are significantly reduced due to increased ion passages through the paracellular route [40]. In addition, we previously showed that tight junction distribution, and, consequently, the permeability of the monolayer was negatively affected by oxidative stress [11]. Therefore, studying the ability of Que and RA to maintain an intact epithelial layer is important. Direct (H_2_O_2_) and indirect (diethyl Maleate) oxidative stress compromise the integrity of the IPEC-J2 monolayer, whereas Trolox and ascorbic acid restore barrier function in the presence of oxidative stress [11]. The present study demonstrated that RA and Que significantly improve the integrity of the IPEC-J2 cell monolayer. Studies using Caco-2 and renal epithelial cells have shown that both RA and Que pre-treatments can protect or upregulate the expression of proteins that make up the tight junction complex, e.g., zonula occludens 1 (ZO-1), ZO-2, claudin-1 and occludin in both unstressed and stressed cells [41,42].

## 5. Conclusions

In conclusion, this study illustrated that RA, Que, GA, ETQ and SeMet have the capacity to increase cellular viability and that RA, Que, ETQ and SeMet decrease intracellular ROS in H_2_O_2_ stressed IPEC-J2 cells. LA was unable to increase viability or decrease iROS in H_2_O_2_ stressed IPEC-J2 cells. Additionally, it was shown that reduction of intracellular oxidative stress can play a role in the preservation of the viability of porcine intestinal epithelial cells and the integrity of the monolayer. Finally, Que and RA proved to be the most promising antioxidants to protect against the detrimental effects of oxidative stress in non-carcinogenic porcine intestinal epithelial cells.
